# Highly Ordered Mesoporous NiCo_2_O_4_ as a High Performance Anode Material for Li-Ion Batteries

**DOI:** 10.3389/fchem.2019.00521

**Published:** 2019-07-23

**Authors:** Qilong Ren, Guangyu Wu, Weinan Xing, Jiangang Han, Pingping Li, Bo Li, Junye Cheng, Shuilin Wu, Rujia Zou, Junqing Hu

**Affiliations:** ^1^State Key Laboratory for Modification of Chemical Fibers and Polymer Materials, College of Materials Science and Engineering, Donghua University, Shanghai, China; ^2^College of Biology and the Environment, Nanjing Forestry University, Nanjing, China; ^3^Department of Vascular Surgery, Shanghai Ninth People's Hospital, Shanghai JiaoTong University School of Medicine, Shanghai, China; ^4^Center of Super-Diamond and Advanced Films, Department of Materials Science and Engineering, City University of Hong Kong, Hong Kong, China

**Keywords:** highly ordered, mesoporous, NiCo_2_O_4_, lithium-ion batteries, nano-casting

## Abstract

The controlled synthesis of highly ordered mesoporous structure has attracted considerable attention in the field of electrochemistry because of its high specific surface area which can contribute the transportation of ions. Herein, a general nano-casting approach is proposed for synthesizing highly ordered mesoporous NiCo_2_O_4_ microspheres. The as-synthesized mesoporous NiCo_2_O_4_ microsphere materials with high Brunner-Emmett-Teller (BET) surface area (~97.77 m^2^g^−1^) and uniform pore size distribution around 4 nm exhibited a high initial discharge capacity of ~1,467 mAhg^−1^, a good rate capability as well as cycling stability. The superior electrochemical performance was mainly because of the highly porous nature of NiCo_2_O_4_, which rendered volume expansion during the process of cycling and shortened lithium-ions transport pathways. These properties showcase the inherent potential for use of highly ordered mesoporous NiCo_2_O_4_ microspheres as a potential anode material for lithium-ion batteries in the future.

## Introduction

Lithium-ion batteries (Yoo et al., 2008; Pan et al., 2013) have been proven to be viable alternatives to traditional energy storage devices and are vital tools when coupled with emerging renewable energy sources (Lewis and Nocera, 2006; Song, 2006; Chheda et al., 2007) (i.e., wind, solar, *etc*.). However, current energy demands outpace what is commercial systems are capable of, leading to the development of next-generation lithium-ion batteries (Liu et al., 2012; Lu et al., 2018; Shen et al., 2018). Particularly, the electrode is vital for optimal electrochemical performance (Toupin et al., 2004; Jiang et al., 2012; Wang et al., 2012; Yang et al., 2019a); however, conventional anode materials (Courtel et al., 2011; Chen et al., 2013; Chang et al., 2014) such as graphite have low theoretical specific capacities (372 mAh g^−1^) and fail to satisfy energy storage demands. Consequently, transition metal oxides (TMOs) (Yuan et al., 2014; Tabassum et al., 2018) have been proposed as viable alternatives, attributed to high energy densities, which result in capacities more than double of those observed with graphite. However, this capacity relies on the morphology and structure of the TMO, which can suffer from undesired volume expansion and collapse of the structure as the battery cycles, leading to catastrophic failure.

Recently, space designed nanostructures have been acknowledged as an effective strategy to remit the volume expansion as well as shorten the lithium-ion transport pathways by providing a larger specific surface area, including implementation of a porous network (Wen et al., 2007; Lou et al., 2008; Shen et al., 2012, 2014; Yuan et al., 2012; Qie et al., 2013; Yang et al., 2019b), mesopores structures. For example, mesoporous Co_3_O_4_ (Li et al., 2008), NiO (Yin et al., 2018), NiCo_2_O_4_ (Li et al., 2018), and SnO_2_ (Han et al., 2019) have been fabricated and have shown good electrochemical performance. However, limited attention has been focused to study ternary systems such as NiCo_2_O_4_ despite a higher electrical conductivity and specific capacity owing to its enhanced chemical kinetics. Inspired by ammonium hydrogen carbonate-assisted solvothermal route to prepare Ni_0.33_Co_0.67_CO_3_ microspheres (Li et al., 2013), they led to a recent breakthrough technique to form mesoporous microspheres, we present a modified structure design containing ordered mesopores to further improve the electrochemical performance of NiCo_2_O_4._

**Figure 1:**
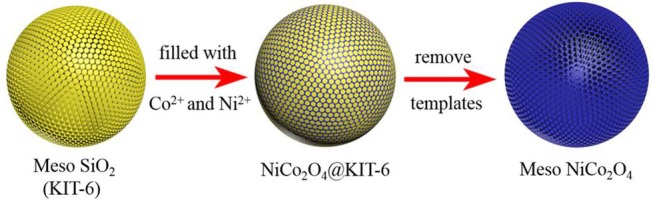
Schematic illustration of the preparation for mesoporous NiCo_2_O_4_ microspheres.

Herein, we developed a template-assisted method to synthesize novel NiCo_2_O_4_ microspheres containing highly ordered mesoporous structures and nanoparticles to effectively alleviate the huge volume expansion and enhance the electrical conductivity. The synthesis process is schematically shown in Figure 1. Firstly, mesoporous silica (KIT-6) is synthesized as a template by a typical approach which is illustrated in the experiment section in detail. Then the template is immersed in the mixed solution of Ni(NO_3_)_2_ and Co(NO_3_)_2_ to introduce the Ni^2+^ and Co^2+^ to be filled into KIT-6. After a subsequent calcination step at 450°C under N_2_ atmosphere, the NiCo_2_O_4_@KIT-6 is successfully prepared. Finally, NaOH solution is utilized to remove the template of KIT-6 in the NiCo_2_O_4_@KIT-6 and obtain mesoporous NiCo_2_O_4_ microspheres. When acted as an anode material for Li-ion batteries, mesoporous NiCo_2_O_4_ microsphere electrode exhibits the superior electrochemical performance, whose stable specific capacity was 430 mAhg^−1^ after 100 cycles, which is better than that of the non-porous NiCo_2_O_4_ (270 mAhg^−1^ after 100 cycles). The improved lithium storage performance mainly benefits from the rationally designed mesoporous structures of NiCo_2_O_4_. We believe that this versatile strategy could be extended to more ternary TMO materials for the development of high property electrode in LIBs.

## Experimental Section

### Materials

All chemicals (analytical reagent grade) used in this work, including polyethylene oxide-polypropylene oxide-polyethylene oxide (PEO-PPO-PEO, P123, MW = 5.8 K), hydrochloric acid, n-butanol, tetraethyl orthosilicate (TEOS), Co(NO_3_)_2_•6H_2_O, Ni(NO_3_)_2_•6H_2_O and ethanol were purchased from Sigma-Aldrich.

### Material Synthesis

#### Synthesis of Mesoporous SiO_2_ (KIT-6)

The typical synthetic process was as follows (Zhou et al., 2018): 4.75 g of P123 were dissolved in a mixed solution containing 163 mL of deionized (DI) water and 7.5 ml of concentrated hydrochloric acid under stirring at 35°C. Once the P123 was fully dissolved, 4.53 g of n-butanol were added. After 1 h, TEOS (9.675 g) were added into the above mixed solution. The solution was then stirred for an additional 24 h at 35°C, followed by another 24 h incubation at 35°C. Cooling the mixed solution to room temperature, the precipitates could be collected by centrifugation for 3 times and moved to a vacuum oven at 90°C for 12 h. Then, after calcining the collected deposit at 550°C for 6 h, white mesoporous SiO_2_ was obtained.

#### Synthesis of Mesoporous NiCo_2_O_4_ Microspheres

In a simple process, 0.4 g of KIT-6 were added to a solution containing 4 ml of 1 M Co(NO_3_)_2_·6H_2_O and 2 ml of 1 M Ni(NO_3_)_2_·6H_2_O in ethanol under stirring for 1.5 h at room temperature. Then, the solution was heated at 70°C until the ethanol was completely evaporated, and the solid was calcined at 200°C for 4 h. Immediately following, 2 mL of 1 M Co(NO_3_)_2_·6H_2_O and 1 mL of Ni(NO_3_)_2_·6H_2_O in ethanol were added following the previous steps and an additional calcination 450°C for 6 h. To obtain mesoporous NiCo_2_O_4_ microspheres, the powder was immersed in a 2 M NaOH solution to etch away the KIT-6 templates. Then, the samples were collected by centrifugation, washed for 3 times and moved to a vacuum oven at 60°C for 12 h. As a control, conventional NiCo_2_O_4_ microspheres were produced through the above-mentioned process without KIT-6 templates.

#### Materials Characterization

The mesoporous NiCo_2_O_4_ microspheres were characterized by using a PANalytical X' Pert X-ray diffractometer (Holland), with Cu-Kα radiation at 40 kV and 40 mA, selected-area electron diffraction (SAED), scanning electron microscope (SEM, S-4800) and transmission electron microscope (TEM, JEM-2100F). The N_2_ adsorption/desorption isotherms were used to calculate the specific surface area and Barrett-Joyner-Halenda (BJH) equation was used to calculate the pore size distribution and average pore diameter.

## Results and Discussion

**Figure 2:**
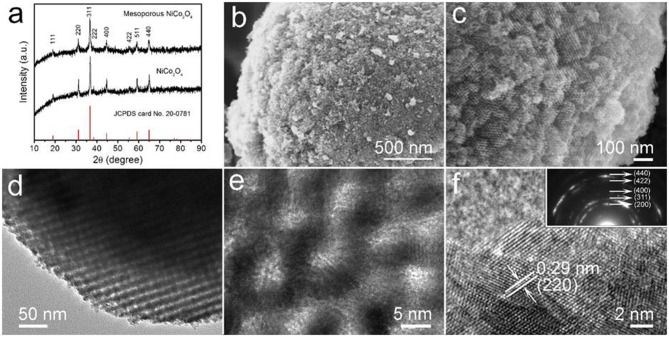
**(a)** XRD patterns of mesoporous and non-porous NiCo_2_O_4_. **(b,c)** high magnification SEM images of mesoporous NiCo_2_O_4_. **(d)** TEM image and **(e,f)** high-resolution TEM image of mesoporous NiCo_2_O_4_. Inset in **(f)** shows the corresponding SAED pattern.

The mesoporous NiCo_2_O_4_ microspheres were prepared via nano-casting, with the crystalline structure and phase purity characterized by X-ray diffraction (XRD). Figure 2a compares the diffraction patterns of both mesoporous and non-porous NiCo_2_O_4_ microspheres, showing eight obvious peaks at 2θ values of 18.9, 31.1, 36.7, 38.4, 44.6, 55.4, 59.1, and 64.9 for the (111), (220), (311), (222), (400), (422), (511), and (440) planes, respectively, which consist with the cubic spinel NiCo_2_O_4_ (JCPDS No.20-0781)without any apparent impurities.

The morphology and structure of obtained NiCo_2_O_4_are elucidated by SEM and TEM, as shown in Figures 2b,c, the surface morphology of the mesoporous microspheres, when compared to nonporous structures (Figure S1), is significantly rougher with highly ordered, uniform pores. This suggests that the polymeric precursor was fully injected within the KIT-6 microspheres, allowing the continuity of the mesoporous structure in subsequent deposition reactions. The highly ordered pores of the NiCo_2_O_4_ microspheres are clearly revealed with a pore size of ~4 nm as shown by high-resolution TEM in Figures 2d,e. Additionally, in Figure 2f, the measured interplanar distance was found to be 0.29 nm, which aligns well with the (220) planes of spinel NiCo_2_O_4_. It is worth noting that well-defined diffraction rings were presented by the SAED pattern (Inset in Figure 2f), which correspond to the (440), (422), (400), (311), and (200) planes. The polycrystalline diffraction rings are in accordance with the result from the XRD pattern.

**Figure 3:**
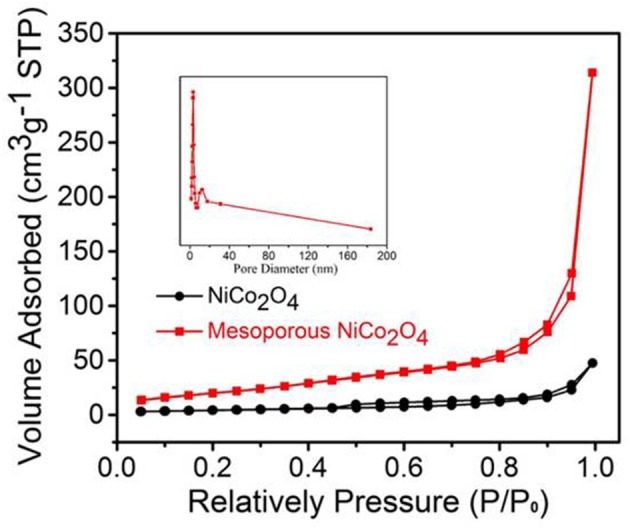
Nitrogen adsorption-desorption isotherm and the corresponding pore size distribution of ordinary NiCo_2_O_4_ and mesoporous NiCo_2_O_4._.

The pore diameter distribution and specific surface area of mesoporous NiCo_2_O_4_ samples were determined via N_2_ adsorption-desorption measurements. The result of specific surface area was calculated from the isotherms (Figure 3) was 97.77 m^2^g^−1^ for mesoporous NiCo_2_O_4_ microspheres, while the non-porous NiCo_2_O_4_ microspheres were 26.63 m^2^g^−1^. Additionally, the mesoporous structure was further analyzed by pore diameter distribution in the inset of Figure 3. NiCo_2_O_4_ microspheres displayed a pore volume (0.494 cm^3^g^−1^) with an average pore diameter of 3.416 nm. Ascribed to this special microsphere structure, the mesoporous structure could shorten the diffusion paths for lithium ions and provided buffering space to adapt the volume expansion during the process of Li^+^ insertion and extraction.

**Figure 4:**
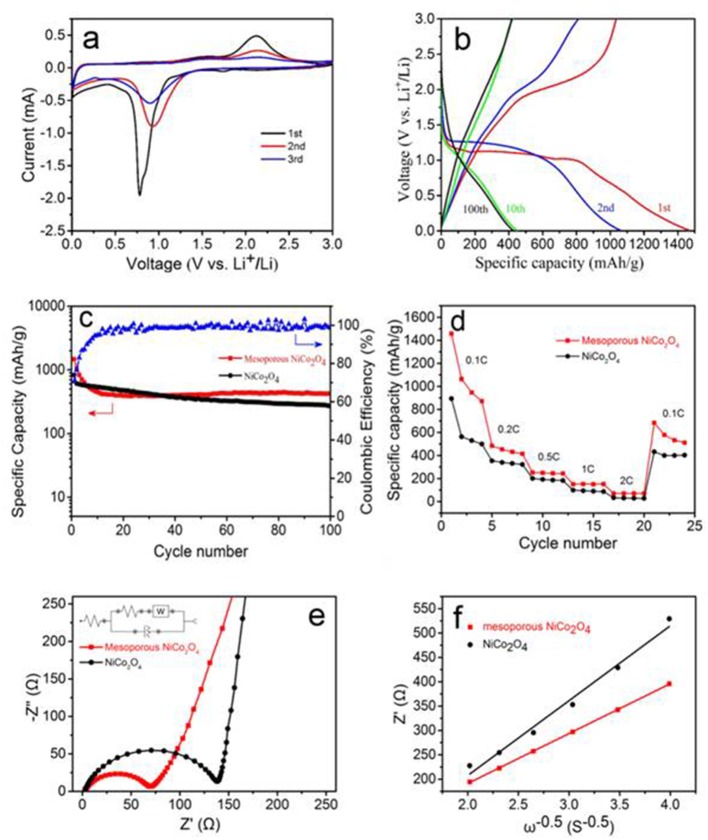
First three consecutive CV curves of mesoporous NiCo_2_O_4_ **(a)**, galvanostatic discharge and charge profiles for 1st, 2nd,10th, 100th cycles of mesoporous NiCo_2_O_4_ **(b)** at the current densities of 100 mA.g^−1^, cycling performance of ordinary NiCo_2_O_4_ and mesoporous NiCo_2_O_4_ at the current densities of 100 mAh.g^−1^ **(c)**, rate performances for ordinary NiCo_2_O_4_ and mesoporous NiCo_2_O_4_ at various current densities **(d)**. EIS pattern of ordinary NiCo_2_O_4_ and mesoporous NiCo_2_O_4_ **(e)**. The typical plots for Z' vs. ω^−0.5^ for the mesoporous NiCo_2_O_4_ **(f)**.

To confirm whether the mesoporous NiCo_2_O_4_ microspheres would be applicable as the anode materials in lithium-ion batteries, the as-prepared products were further investigated using cyclic voltammetry (CV), where Figure 4a exhibits the first three cycles of the mesoporous NiCo_2_O_4_ microspheres. In the 1st cycle, one dominant peak at 0.8 V could be assigned to the decomposition and reduction of Ni and Co ions. Meanwhile, the anodic peaks at approximately 2.1 V could be owing to the oxidation reaction of metallic Ni and Co to NiO and CoO. In subsequent tests, the cathodic peak broadened and shifted to 1.0 V. According to the analysis of the CV curves and previous literature reports, the Li reactions for mesoporous NiCo_2_O_4_ materials are as follows:

(1) $$\begin{array}{rcl} {\text{NiCo}}_{2}O_{4} & + & 8{\text{Li}}^{+}+8e^{-}=\text{Ni}+2\text{Co}+4{\text{Li}}_{2}O \end{array}$$

(2) $$\begin{array}{rcl} \text{Ni} & + & {\text{Li}}_{2}\text{O}=\text{NiO}+2{\text{Li}}^{+}+2{\text{e}}^{-} \end{array}$$

(3) $$\begin{array}{rcl} 2\text{Co} & + & 2{\text{Li}}_{2}\text{O}=2\text{CoO}+4{\text{Li}}^{+}+4{\text{e}}^{-} \end{array}$$

(4) $$\begin{array}{rcl} 2\text{CoO} & + & 2/3{\text{Li}}_{2}\text{O}=2/3{\text{Co}}_{3}{\text{O}}_{4}+4/3{\text{Li}}^{+}+4/3{\text{e}}^{-} \end{array}$$

Galvanostatic tests were executed to ensure the influence of highly ordered mesoporous NiCo_2_O_4_ samples on the specific capacity values and capacity retention. The 1st, 2nd, 10th, and 100th cycles of the galvanostatic charge and discharge curves for mesoporous samples, at a current of 100 mA.g^−1^, are shown in Figure 4b. A long voltage plateau was showed in the initial discharge curve between 0.7 and 1.2 V, corresponding to the strong reduction peak that appeared during the first cathodic CV scan. The initial charge capacity for the mesoporous NiCo_2_O_4_ microspheres was ~1467 mAhg^−1^, which is markedly higher than the non-porous NiCo_2_O_4_ microspheres (~820 mAhg^−1^, Figure S2); however, this decreased to 1,060 mAhg^−1^ after the 2nd discharge. The irreversible capacity increase during the 1st charge may be due to the generation of a solid electrolyte interface (SEI) layer.

A comparison of the specific capacity values obtained for non-porous NiCo_2_O_4_ and mesoporous NiCo_2_O_4_ over 100 cycles plotted is shown in Figure 4c. The non-porous NiCo_2_O_4_ suffers the most severe capacity fading with capacity values decreasing to 270 mAhg^−1^ after 100 cycles, while the capacity retention was improved for mesoporous NiCo_2_O_4_ microspheres, obtaining capacity values of 430 mAhg^−1^ after 100 cycles. The significant increase in the capacities for mesoporous NiCo_2_O_4_ compared to ordinary NiCo_2_O_4_ is attributed to the inherent properties of the mesoporous structure. Nevertheless, the final capacity is relatively low compared to the initial capacity, which may be attributed to the aperture being too small to relieve the expansion of the active material completely (Bhaway et al., 2017). For conversion-mode materials, the mesoporous NiCo_2_O_4_ provides particularly high buffering space, to adapt the large volume expansion during the process of Li^+^ insertion/extraction and shorten the diffusion paths for lithium ions due to the interconnected architectures (Figure S3). Moreover, the coulombic efficiency for mesoporous NiCo_2_O_4_ over 100 cycles is also shown in Figure 4c. The initial coulombic efficiency is quite low (~70%); however, the efficiency remains >95% after the 10th cycle. The rate performance of ordinary NiCo_2_O_4_ microspheres and mesoporous NiCo_2_O_4_ microspheres at various current densities were then compared in Figure 4d. The mesoporous NiCo_2_O_4_ microsphere electrode exhibits high initial discharge capacity and the capacity was recession with the increase of current density. However, the mesoporous NiCo_2_O_4_ microsphere electrode exhibited a higher capacity and rate of lithium-ion storage than the non-porous NiCo_2_O_4_ microsphere electrode.

To further investigate the mechanism for the improved electrochemical performance of the mesoporous NiCo_2_O_4_ microsphere, electrochemical impedance measurements (Figure 4e) and a fitting process (Figure S4) were measured containing the mesoporous NiCo_2_O_4_ electrode and ordinary NiCo_2_O_4_. As shown in Figure 4e, a semicircle and a straight line were acquired in the high frequency part and low frequency part, respectively. The intersection point from the curve in the high frequency part is on behalf of the electrolyte resistance. Moreover, the high frequency semicircle corresponds to the charge transfer resistance and the low frequency region with an inclined line represents the process of Li-ion diffusion. The initial value for charge transfer resistance is 66 Ω, which is far less than the ordinary NiCo_2_O_4_ (134 Ω, Table S1), which could be related to the more effective ion and electron transfer in the interface of electrolyte and active material, so that the cell has improved electrode reaction kinetics and a better cell cycling results.

Moreover, the diffusion coefficient of the lithium ions (D${}_{\text{Li}}^{+}$) can be confirmed in accordance with the following equation:

$$\begin{array}{l} {\text{D}}_{{\text{Li}}^{+}}=\frac{0.5{\text{R}}^{2}{\text{T}}^{2}}{{\text{A}}^{2}{\text{F}}^{4}{\text{c}}^{2}\sigma_{\omega }^{2}} \end{array}$$

Where R = gas constant, T = absolute temperature, A = surface area of the electrode, F = Faraday constant, c = concentration of Li+ in the material, and σ = Warburg factor obeying the following relationship:

$$\begin{array}{l} {\text{Z}}_{Re}={\text{R}}_{\text{S}}+{\text{R}}_{\text{ct}}+\sigma \omega^{-0.5} \end{array}$$

Where Z_Re_ is the real part of impedance, thus ω is the angular frequency at low frequency. The linear relationship of Z_Re_ and ω^−0.5^ is shown in Figure 4f, with the slope of the fitted straight line indicating the value of σ. Table 1 revealed the calculated values of σ and D${}_{\text{Li}}^{+}$, where it can be clearly seen that the mesoporous NiCo_2_O_4_ exhibits a higher value of D${}_{\text{Li}}^{+}$ than nonporous NiCo_2_O_4_.

**Table 1:** Warburg factor (σ) and diffusion coefficient ${\text{(D}}_{\text{Li}}^{\text{+}}\text{)}$ of sample ordinary NiCo_2_O_4_ and mesoporous NiCo_2_O_4_.

**Materials**	**σ(Ω s^**−0.5**^)**	**${\text{D}}_{\text{Li}}^{\text{+}}$ (cm^**2**^s^**−1**^)**
Ordinary NiCo_2_O_4_	153.78	1.64 ×10^−12^
Mesoporous NiCo_2_O_4_	102.48	2.46 ×10^−12^

## Conclusion

Highly ordered mesoporous NiCo_2_O_4_ microspheres with a honeycomb-like structure, were synthesized via a nano-casting method. The mesoporous NiCo_2_O_4_ electrode possesses a high initial discharge capacity of ~1,467 mAh.g^−1^ at 100 mAg^−1^ and it exhibited both a high surface area and good rate capability. Such high electrochemical performance is due to its excellent surface area of mesoporous NiCo_2_O_4_ and the rapid ion transport in the electrolyte/electrode interface. This work may open a new sight in the synthesis of excellent Li-storage electrode materials and show an application in a promising candidate for Li-ion batteries.

## Data Availability

The raw data supporting the conclusions of this manuscript will be made available by the authors, without undue reservation, to any qualified researcher. Requests to access the datasets should be directed to hu.junqing@dhu.edu.cn.

## Author Contributions

GW, BL, and JHu designed the project. QR, WX, JHa, PL, JC, SW, and RZ carried out the experiment and performed the experimental data analysis. QR, GW, and WX wrote the paper. BL revised the manuscript. All authors contributed to discussion of the results.

### Conflict of Interest Statement

The authors declare that the research was conducted in the absence of any commercial or financial relationships that could be construed as a potential conflict of interest.
