# Corrigendum: Highly Ordered Mesoporous NiCo_2_O_4_ as a High Performance Anode Material for Li-Ion Batteries

**DOI:** 10.3389/fchem.2019.00600

**Published:** 2019-08-28

**Authors:** Qilong Ren, Guangyu Wu, Weinan Xing, Jiangang Han, Pingping Li, Bo Li, Junye Cheng, Shuilin Wu, Rujia Zou, Junqing Hu

**Affiliations:** ^1^State Key Laboratory for Modification of Chemical Fibers and Polymer Materials, College of Materials Science and Engineering, Donghua University, Shanghai, China; ^2^College of Biology and the Environment, Nanjing Forestry University, Nanjing, China; ^3^Department of Vascular Surgery, Shanghai Ninth People's Hospital, Shanghai JiaoTong University School of Medicine, Shanghai, China; ^4^Center of Super-Diamond and Advanced Films, Department of Materials Science and Engineering, City University of Hong Kong, Hong Kong, China

**Keywords:** highly ordered, mesoporous, NiCo_2_O_4_, lithium-ion batteries, nano-casting

In the original article, the author order list was incorrectly showed as Guangyu Wu^1*†^, Qilong Ren^2†^, Weinan Xing^2*^, Jiangang Han^2^, Pingping Li^2^, Bo Li^3*^, Junye Cheng^4^, Shuilin Wu^4^, Rujia Zou^1^, Junqing Hu^1*^. The correct author order's list is Qilong Ren^1†^, Guangyu Wu^2*†^, Weinan Xing^2*^, Jiangang Han^2^, Pingping Li^2^, Bo Li^3*^, Junye Cheng^4^, Shuilin Wu^4^, Rujia Zou^1^, Junqing Hu^1*^.

In the published article, reflecting the above correction, there was an error with affiliations “1” and “2”. Instead of appearing as “1 College of Biology and the Environment, Nanjing Forestry University, Nanjing, China” and “2 State Key Laboratory for Modification of Chemical Fibers and Polymer Materials, College of Materials Science and Engineering, Donghua University, Shanghai, China,” they should be reversed to appear as “1 State Key Laboratory for Modification of Chemical Fibers and Polymer Materials, College of Materials Science and Engineering, Donghua University, Shanghai, China” and “2 College of Biology and the Environment, Nanjing Forestry University, Nanjing, China”.

Furthermore, there was an error in affiliation “4”. Instead of “Department of Materials Science and Engineering, Center of Super-Diamond and Advanced Films, City University of Hong Kong, Hong Kong, China,” it should be “Center of Super-Diamond and Advanced Films, Department of Materials Science and Engineering, City University of Hong Kong, Hong Kong, China”.

The authors apologize for this error and state that this does not change the scientific conclusions of the article in any way. The original article has been updated.

