# Supplementary material for: Highly Ordered Mesoporous NiCo2O4 as a High Performance Anode Material for Li-Ion Batteries
Source: Front Chem. 2019 Jul 23;7:521. doi: 10.3389/fchem.2019.00521 (PMC6664485; doi:10.3389/fchem.2019.00521)
Supplement: Supplementary file 1 [file Data_Sheet_1.docx]

Supplementary Material

Highly Ordered Mesoporous NiCo_2_O_4_ as a High Performance Anode Material for Li-ion Batteries

**Guangyu Wu1*†, Qilong Ren2†, Weinan Xing2*, Jiangang Han2, Pingping Li 2, Bo Li 3*,**

Junye Cheng4, Shuilin Wu4, Rujia Zou1 and Junqing Hu1*

*1 College of Biology and the Environment, Nanjing Forestry University, Nanjing, China,*

*2 State Key Laboratory for Modification of Chemical Fibers and Polymer Materials, College of Materials Science and Engineering, Donghua University, Shanghai, China,*

*3 Department of Vascular Surgery, Shanghai Ninth People’s Hospital, Shanghai JiaoTong University School of Medicine, Shanghai, China,*

*4 Department of Materials Science and Engineering, Center of Super-Diamond and Advanced Films, City University of Hong Kong, Hong Kong, China*

*** Correspondence:**

Guangyu Wu：gywuchem@163.com

Weinan Xing: xingweinan@126.com

Bo Li: boli@shsmu.edu.cn

Junqing Hu: hu.junqing@dhu.edu.cn

**1. Electrochemical Measurements**

The electrochemical performances of mesoporous NiCo_2_O_4_ microspheres were carried out using coin cells (CR2032), in which lithium was used as the counter electrode and reference electrode. The working electrode was constituted by the (70 Wt %) mesoporous NiCo_2_O_4_ microspheres, (20 Wt %) acetylene carbon black and (10 Wt %) polyvinylidene fluoride (PVDF). After stirring, the mixed materials were pasted uniformly on to the copper foil substrate. Finally, this electrode was moved to a vacuum oven at 60 °C overnight. The microporous polythene (Celgard2400) was used as a separator, and the electrolyte employed was 1 M LiPF6 in ethylene carbonate and diethyl carbonate. The test cells were assembled in an argon-filled glovebox (UniLab, MBRAUN, Germany). The galvanostatic charge-discharge cycling was carried out using NEWARE-BTS battery tester with a potential window of 0.01-3.0V at room temperature. All the cells were measured on an Autolab (PGSTAT302N) electrochemistry workstation.

# 2. Supplementary Data

**
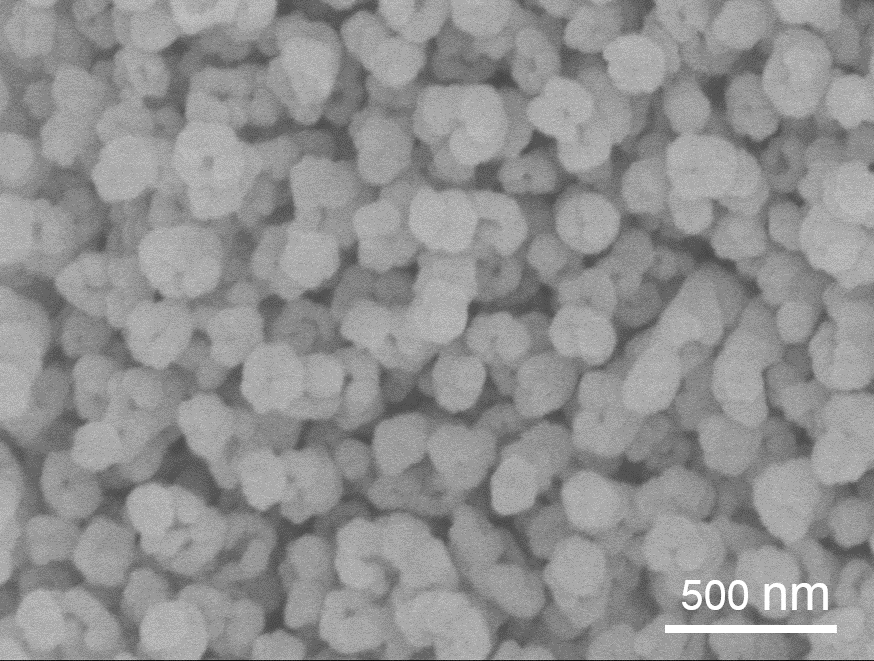
**

**Figure S1**. low-resolution SEM image of ordinary NiCo_2_O_4_.


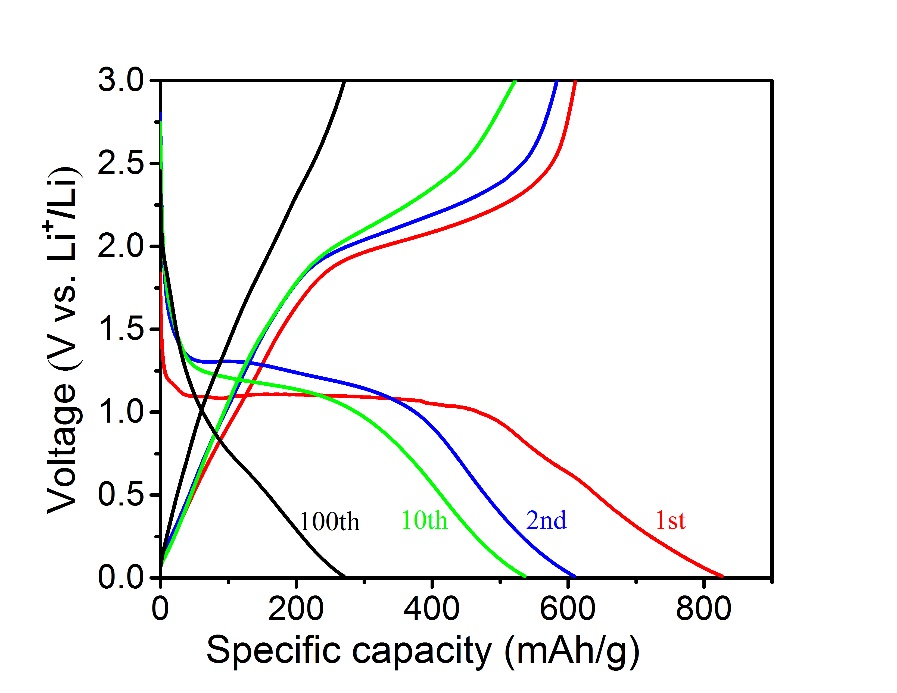


**Figure S2**. Galvanostatic discharge and charge profiles for 1st, 2nd, 10th, 100th cycles of ordinary NiCo_2_O_4_ at the current densities of 100 mA.g^-1^.


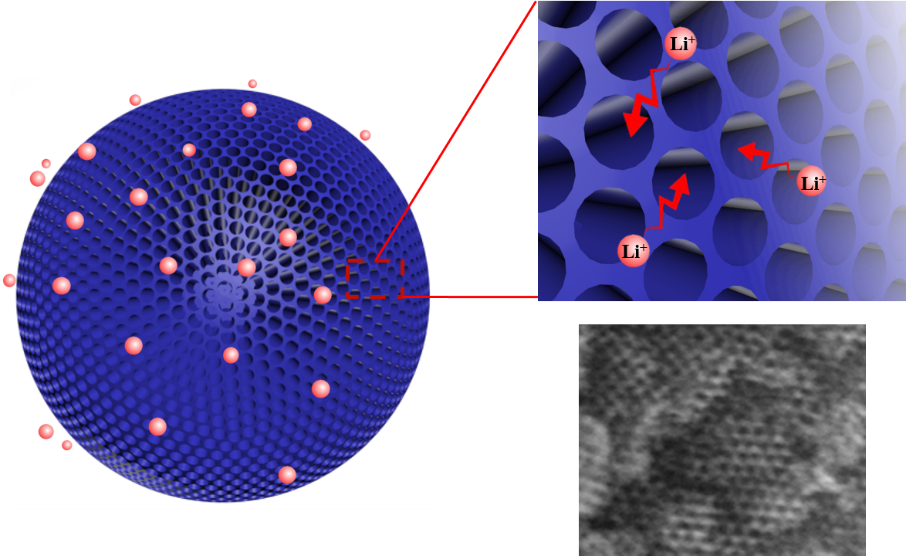


**Figure S3**. Schematic illustration of the 3D mesoporous network of ion transport

pathways and TEM image of its pore. The scale bar is 10nm.


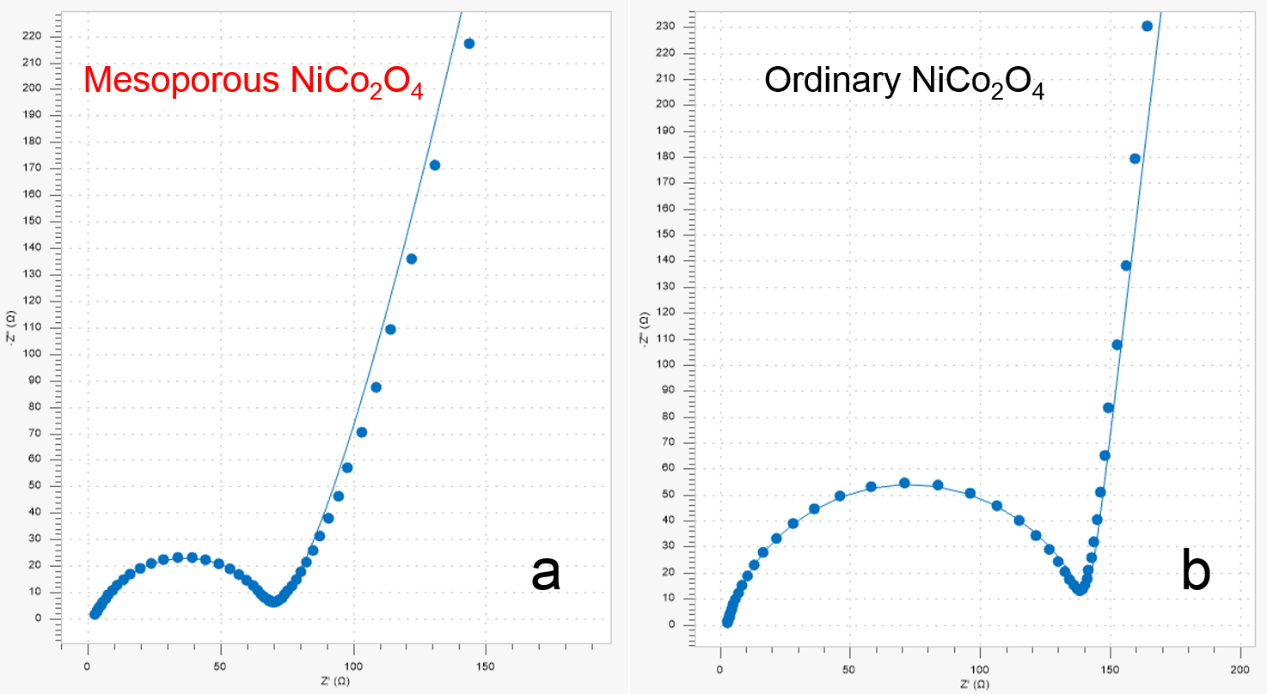


**Figure S4**. EIS spectra of (a) mesoporous NiCo_2_O_4_, (b) ordinary NiCo_2_O_4_ electrodes that contain the fitting curves.

| **Table S1**: Electrochemical impedance spectroscopy (EIS): initial resistance (Rs) and charge transfer resistance (Rct). |
| --- |
| Materials Rs (Ω) Rct (Ω) |
| Ordinary NiCo_2_O_4_ 2.5 134  Mesoporous NiCo_2_O_4_ 1.8 1 66 |
|  |
